# Target Product Profile (TPP) for Chagas Disease Point-of-Care Diagnosis and Assessment of Response to Treatment

**DOI:** 10.1371/journal.pntd.0003697

**Published:** 2015-06-04

**Authors:** Analía I. Porrás, Zaida E. Yadon, Jaime Altcheh, Constança Britto, Gabriela C. Chaves, Laurence Flevaud, Olindo Assis Martins-Filho, Isabela Ribeiro, Alejandro G. Schijman, Maria Aparecida Shikanai-Yasuda, Sergio Sosa-Estani, Eric Stobbaerts, Fabio Zicker

**Affiliations:** 1 Pan American Health Organization, Regional Office of the World Health Organization, Washington, D.C., United States of America; 2 Servicio de Parasitología y Chagas, Hospital de Niños Ricardo Gutiérrez, Ciudad de Buenos Aires, Argentina; 3 Laboratório de Biologia Molecular e Doenças Endêmicas, Oswaldo Cruz Institute, Laboratory of Molecular Biology and Endemic Diseases, FIOCRUZ, Rio de Janeiro, Brazil; 4 Sergio Arouca National School of Public Health, FIOCRUZ, Rio de Janeiro, Brazil; 5 Médecins Sans Frontières—Médecins Sans Frontières Operational Center Barcelona-Athens (OCBA), Barcelona, Spain; 6 René Rachou Research Center, Laboratory of Biomarkers of Diagnostic and Monitoring, Oswaldo Cruz Institute, Minas Gerais, Brazil; 7 Latin America Regional Office, Drugs for Neglected Diseases initiative, Rio de Janeiro, Brazil; 8 Laboratorio de Biología Molecular de la Enfermedad de Chagas, Instituto de Investigaciones en Ingeniería Genética y Biología Molecular “Dr Hector Torres” (INGEBI-CONICET), Buenos Aires, Argentina; 9 Department of Infectious and Parasitic Diseases, Faculdade de Medicina, University of São Paulo, Sao Paulo, Brazil; 10 Instituto Nacional de Parasitología, Dr. Mario Fatala Chaben ANLIS, Ministerio de Salud, Buenos Aires, Argentina; 11 Center for Technological Development in Health, Oswaldo Cruz Foundation, (FIOCRUZ), Rio de Janeiro, Brazil; US Food and Drug Administration, UNITED STATES

## Introduction

Chagas disease, or American trypanosomiasis, affects 8 million people, largely in Latin America, where it is endemic in all countries. With an overall estimate of 65 million people at risk of contracting the disease, 28,000 new cases every year, and 12,000 deaths annually, Chagas disease is the most important parasitic disease in the Americas [1]. In addition, nonendemic countries such as the United States [2,3], Canada [4], Germany [5], Italy [6], Spain [7,8], Switzerland [9,10], and France [11] have experienced the occurrence of *Trypanosoma cruzi–*infected and Chagas disease cases; the majority of these cases are among immigrants coming from endemic Latin American countries [12]. Like other neglected tropical diseases (NTDs), Chagas disease affects mostly poor populations with limited access to health services. Vector transmission is associated with poor housing in periurban and rural areas. After infection, the disease is characterized by an acute phase, usually asymptomatic, which evolves in 20%–30% of the patients to a chronic disabling cardiac and/or digestive clinical form. The remaining infected individuals evolve to a chronic asymptomatic but infective clinical phase [13]. Reactivation of chronic Chagas disease may occur associated with comorbidities such as HIV/AIDS, organ transplants, or immunosuppressive therapy [14].

Following resolutions from the Pan American Health Organization (PAHO) and World Health Organization (WHO) on the prevention, care, and control of Chagas disease, several countries in the Americas have strengthened control activities and achieved significant progress towards this goal. Yet, the disease remains prevalent among marginalized populations in the continent; many patients remain undiagnosed and untreated, making Chagas disease the NTD with the highest burden in Latin America countries. In addition, infected and diseased individuals are increasingly diagnosed in different parts of the world [15].

Case management and treatment are essential strategies to eliminate Chagas disease as a public health problem. However, ensuring diagnosis and access to treatment for millions of infected people continues to be a challenge. Timely diagnosis and trypanocidal treatment are known to reduce the likelihood of disease progression and to prevent congenital transmission. Indeed, Chagas disease has been categorized as “the most neglected of the neglected diseases,” with lingering research and development gaps related to its treatment and diagnosis [16].

During the acute phase, diagnosis relies primarily on direct parasitological tests and secondarily on serological testing. Alternatively, during the chronic phase, diagnosis relies primarily on serological tests and secondarily on molecular tests, which are not readily available in primary health centers outside urban areas. Currently available diagnostic methods and medicines, however, are considered suboptimal for adequate control and treatment programs, reflecting the lackluster investment devoted to this disease [17]. At this time, the two available treatments, nifurtimox and benznidazole, are largely considered to be effective in the acute and early chronic infection and in preventing congenital transmission in children born to infected and treated mothers [18]. While there is a growing body of information to support the use of these medicines in the later stages of the disease, the effectiveness of these drugs outside the acute phase and the safety profile of these treatments need to be further established. Treatment is contraindicated during pregnancy, severe renal or hepatic insufficiency, and severe granulocitopenia and aplastic anemia in immunosuppressed patients, and thus, newer medicines with better risk-benefit profiles are necessary [17,19]. For diagnosis, there is a lack of effective tools for large-scale screening, point-of-care diagnosis, and monitoring patient’s response to antiparasitic treatment. In the case of asymptomatic acute infections, active search is preferred, involving direct parasitological methods and serology (immunoglobulin M [IgM] antibody anti-*T*. *cruzi*) for contacts or suspects. Serological tests are not considered to be reliable in endemic areas for diagnosis of acute asymptomatic infection because of the absence of good standardized commercial kits. Standard diagnostic protocols are hard to implement outside of large urban centers where one can anticipate a large number of infected individuals.

The complexity of the equipment that is required, the need for highly trained personnel, and the need for the patient to come to the health center more than once are among some of the most pressing constraints. Moreover, serodiagnosis in infants born to seropositive mothers has low positive predictive value due to the passive transfer of maternal anti-*T*. *cruzi* IgGs. At the same time, a small but sizeable number of infected newborns may be seronegative. Thus, it is recommended to conduct parasite search in this population using microhematocrit, hemoculture (HC), and PCR; none of these tests are easily performed outside laboratories performing tests of moderate and high complexity. Hence, developing new diagnostic tools that are easy to use and adapted to the needs of affected populations and to the reality of health systems based on primary health care will greatly improve the ability to control Chagas disease in the Americas [16,20].

A first and critical step to address the research and development gap is to establish a consensus on the desirable product profiles in different conditions of use. To foster and inform the development of these much needed tools, PAHO, in collaboration with the Drugs for Neglected Diseases initiative (DNDi), Médecins sans Frontières (MSF), and the Special Programme for Research and Training in Tropical Diseases (TDR), convened a multidisciplinary group of experts in Rio de Janeiro, Brazil (April 2010), to review the evidence and initiate discussions. The meeting established the basis for the development of target product profiles (TPPs), which are reported in this paper.

## Target Product Profile Consensus

The working group proposed TPPs for three different scenarios:

1. point-of-care diagnosis for patients in the acute phase (associated with congenital, vector, oral, transplant, or transfusion transmission and infection reactivation) (Table 1);
2. point-of-care diagnosis for asymptomatic or symptomatic patients in the chronic phase (Table 2); and
3. assessment of response to antiparasitic treatment in the chronic phase (Table 3).

The group defined the following critical attributes for the different diagnostics methods according to the three specific clinical scenarios:

1. current need: as it relates to individual care, population-based programs, and situation of use;
2. medical conduct: why and how the diagnostic tool will be applied;
3. sampling: biological material, volume, preservation, and transportation process;
4. infrastructure needed: equipment, shipping procedures, and temperature control;
5. number of samples recommended per test and fractionation;
6. technical skills to perform the test;
7. testing site and turnaround time: time from obtaining samples to produce results;
8. test reading: as qualitative or quantitative;
9. taxonomic diagnosis: the capacity to differentiate parasite strains;
10. sensitivity: the ability to correctly identify all positive cases expressed in percentage; and
11. specificity: the ability to correctly identify all negative cases expressed in percentage (and the risk of cross-diagnosis of other prevalent diseases)

Establishing a TPP for the diagnostics and treatment monitoring of *T*. *cruzi* infection is an important step to guide research and development efforts.

**Table 1:** TPP for point-of-care diagnosis for patients in the acute phase of Chagas disease.

Needs for Diagnosis	Medical Conduct	Samples and Sampling	Infrastructure	Technical Skills	Testing Site, Turnaround Time	Reading	Taxonomic Diagnosis	Sensitivity	Specificity
Congenital transmission	Serodiagnosis of pregnant women and women admitted at delivery living or born in endemic countries (knowing that >70% have no signs or symptoms)	Samples processed individually. (i) Maximum. 2 ml of cord or peripheral blood obtained specifically for diagnostic test; (ii) Blood sample collected for routine screening for infectious or metabolic diseases; (iii) **Ideal:** urine sample	(i) **Ideal:** processing at point of care; (ii) **Less desirable:** samples processed in a reference laboratory transported without cold chain	Good laboratory practices (GLP)–trained technical staff with quality certification. Screening conducted by staff who assisted the childbirth	Primary health centre (PHC), hospital or delivery institution. **Ideal timing:** <1 h, up to a maximum of 12 h from sampling	Qualitative	Single universal test should detect all circulating strains	>95%	100%. **Ideal:** integrated into routine health care screening (e.g., metabolic screening)
Vector and oral transmission	(i) Differential diagnosis for at risk population with febrile syndrome; (ii) Active search in cases of possible exposure (contacts)	Samples processed individually. (i) 2–5 mL blood or serum; (ii) **Ideal:** urine, saliva sample	(i) **Ideal:** processing at point of care; (ii) **Less desirable:** samples processed in a reference lab and transported without cold chain	GLP-trained technical staff with quality certification	PHC and/or community-based diagnosis facility. **Ideal timing:** <1 h from sampling	Qualitative/quantitative	Single universal test should detect all circulating strains	>95%	100%. **Ideal:** integrated into routine health care screening (e.g., metabolic screening). Should differentiate *T*. *cruzi* from *T*. *rangeli*
Reactivation of infection associated with immune suppression in organ transplants. Blood transfusion transmission	Active surveillance	Samples processed individually. Blood, cerebral spinal fluid, tissue from chagoma	(i) **Ideal:** processing at point-of-care; (ii) **Less desirable:** samples processed in a reference laboratory and transported without cold chain	GLP-trained technical staff with quality certification	Reference medical facility, blood banks, and hospital. **Ideal timing:** <1 h from sampling	Qualitative/quantitative	Single universal test should detect all circulating strains	>95%	100%. **Ideal:** integrated into routine screening. Should differentiate between *T*. *cruzi* and *T*. *rangeli*. In the case of diagnosis in central nervous system manifestations of HIV/AIDS, it should differentiate *T*. *cruzi* from other opportunistic infections, such as toxoplasmosis.

**Table 2:** TPP for point-of-care diagnosis for patients in chronic phase of Chagas disease.

Needs for Diagnosis	Medical Conduct	Samples and Sampling	Infrastructure	Technical Skills	Testing Site, Turnaround Time	Reading	Taxonomic Diagnosis	Sensitivity	Specificity
Asymptomatic infected patients, referred symptomatic individuals, and positive blood donors	Active search in endemic/nonendemic and remote areas; prenatal screening	Samples processed individually. **Ideal:** saliva/urine; **Alternative:** whole blood, plasma or serum	Point of care, including community-based facility external to health center (no transportation required)	Adequately trained technical staff or community works with minimum quality certification standards	PHC and community setting (home, school, or community center); **Ideal timing**: <1 h from sampling	Qualitative	Single universal test should detect all circulating strains	Equal to or greater than standard serological tests	100%. No cross-reaction with other parasites (e.g., Leishmania, *T*. *rangeli*)

**Table 3:** Assessment of response to anti-parasitic treatment in the chronic phase.

Needs for Diagnosis	Medical conduct	Samples and Sampling	Infrastructure	Technical Skills	Test Site	Reading	Taxonomic Diagnosis	Sensitivity	Specificity
Assess antiparasitic therapeutic response (based on persistent negativization of parasitemia or reduced parasitic load evaluation though molecular biology methods)^1^	Direct or indirect demonstration of the presence of the parasite in blood or tissue: (i) Before, during, and after treatment (end point >12 months); ii) Therapeutic failure (through the presence of the parasite or parasitic DNA/ antigens in blood)	2–3 samples (before and after treatment), **Ideal:** blood (maximum of 5 mL [adults] and 2 mL [children]); **Ideal:** urine	Reference center, PHC and second level of care. **Ideally:** no cold chain; **Acceptable:** minimum cold chain (2°C–8°C); **Unacceptable:** conservation < 0°C (freezer)	GLP-trained technical staff with-quality certification	Any health facility accessible and convenient for the patient; **Maximum time for result:** 1 week	Quantitative/qualitative	**Ideal:** taxonomic diagnosis (in the case of therapeutic failure); **Acceptable:** detects all circulating strains, but no taxonomic diagnosis	>95%	100%

^1^There is no consensus on the definition of cure, but experts agree that the persistent negativization of parasitemia is the most appropriate marker [23].

In this paper, the TPPs are proposed according to the demands and needs of different population groups and today’s diagnostics and treatment-monitoring challenges. These TPPs represent ideal features of a test adapted to the usual medical conditions in endemic countries. The proposed profiles take into consideration the ease of obtaining samples and the difficulties of transportation and processing them at the point of care, as well as the required infrastructure and the skills needed for performing the test. Irrespective of its specific characteristics and potential uses, the following attributes should be common to any ideal Chagas diagnostic test for public health use:

- Low cost, sustainable production and supply requirements, and based on simple manufacturing and distribution methods
- The diagnostic kits should contain all necessary materials for obtaining the sample and performing the test
- All reagents and other kit materials should be stable in prevalent climatic conditions
- Ease of disposal in accordance with standards of biosecurity
- Acceptability by the health system and target population

The consensus of the group is summarized in Tables 1–3.

## Discussion

Access to appropriate diagnostic tools is critical for individual care and public health control of Chagas disease. A series of publications have reviewed the current challenges related to diagnosis and monitoring of treatment response in patients affected with this disease [16,17,19,20–22]. Many have pointed to the need for an effective and affordable point-of-care test that can be used in endemic areas and administered by low-skilled health workers. This would greatly improve the detection of Chagas disease infections and facilitate appropriate treatment for affected populations, thus reducing the burden to individuals and to the health care system.

Detection of circulating parasites through a series of direct or indirect assays is the approach of choice for diagnostics during the acute phase of Chagas disease. In the acute phase, antiparasitic treatment can eliminate parasites and prevent chronic phase complications. A large proportion of patients remain undiagnosed at this stage, however, because of subclinical presentation of infection and limited access of the affected populations to the health system. Moreover, diagnosis of congenital Chagas disease at the time of birth is considered ideal for timely and effective treatment, particularly in rural areas, since it is difficult for the mothers to take their children for confirmation of diagnosis at 10 months of age. However, there is some evidence showing that parasitic loads may increase during the first month of life and sensitivity of parasitological methods might be better at this period [23]; thus, more studies to assess cost-benefit of this issue are still needed.

In addition, new tools are needed for situations such as oral transmission outbreaks. These facts underline the importance of new diagnostic methods that can inform medical and public health decision-making in endemic countries. Indirect serological methods are currently used for diagnosis during the chronic or silent stage of the disease. A negative seroconversion has been used to assess treatment response, but it may involve a long follow-up period, in particular for chronically infected patients, which is impractical for public health interventions. There is a renewed interest in measuring treatment response to antiparasitic drugs during the chronic phase. While the treatment goal for infectious diseases is or should be pathogen elimination, there are other equally important therapeutic outcomes to be considered [24]. For some infections, such as AIDS, control and reduction of the pathogen burden are well-recognized strategies for converting a fatal disease into a chronically controlled disease, with the administration of appropriate treatments. Recent clinical trials may provide definitive evidence that patients with chronic phase Chagas disease can also benefit from treatment with antiparasitic medicines [21,24]. Current clinical trials during the indeterminate phase or in chronic cardiac Chagas disease patients are based on quantitative PCR (qPCR) methods to monitor the levels of circulating parasites and to detect therapeutic response. Although molecular based techniques are proven useful in a clinical trial setting [25], it is hard to envision their use, as they stand today, in the follow-up of extensive populations in the field. A necessary next step is the development of commercial kits based on recently standardized methods such as real-time PCR [25,26]. Other nucleic acid amplification methods such as loop-mediated isothermal amplification are currently under standardization [27]. However, these kits might have a more substantial impact on case management in more developed countries. Studies are under way to assess the negative predictive value of a PCR test to assess sterile cure (Isabela Ribeiro, personal communication). A recent publication [23] shows that there are several candidate markers, which together may fulfill acceptable criteria to indicate the efficacy of trypanocidal treatment.

Data from ongoing studies are considered essential to improve assessment of existing markers and to identify those for early follow-up of treated patients [23]. Thus, developing a more straightforward technique to directly or indirectly monitor parasitemia in chronic patients that is easier to standardize for wider use in clinical settings is necessary.

Innovative and promising strategies are currently being tested that may contribute to the development of new diagnostic tools for Chagas disease and that comply with several of the criteria included in the proposed TPP. In particular, the tests foreseen to be used at the primary health centre (PHC) or community level (as noted in column 6 of Tables 1 and 2) should comply with the ASSURED criteria [22]: (1) affordable by those at risk of infection; (2) sensitive (few false negatives); (3) specific (few false positives); (4) user friendly (simple to perform and requiring minimal training); (5) rapid (to enable treatment at first visit) and robust (does not require refrigerated storage); (6) equipment-free; and (7) delivered to those who need it. For other settings, more complex technologies such as enzyme-linked immunosorbent assay (ELISA) and PCR might be acceptable. Molecular methods applied to dried blood on filter paper, which has been carried out for triatomine samples [28,29], could enhance detection accuracy in acute and congenital Chagas disease cases. Moreover, serological methods based on low-volume samples are currently under standardization and validation [30]. Recently, a nanoparticle-based assay using urine as the clinical sample has been developed for diagnosis of congenital Chagas disease [31].

Novel nucleic acid amplification approaches such as loop-mediated isothermal amplification, which are currently undergoing standardization and validation for other trypanosomatide infections, should be explored in Chagas disease diagnosis [32,33]. While flow cytometry has been shown to be a good platform, with high sensitivity and specificity for Chagas disease diagnosis and post-therapeutic cure monitoring [34,35], the use of flow cytometry-based devices to develop "point-of-care" TTPs does not seem to be a feasible approach, mainly considering the complexity and the nonportable nature of these devices.

The elimination of Chagas disease in the Americas requires long-term commitments and multidimensional strategies. Development of more appropriate diagnostic tools and treatment options can certainly accelerate and improve the chances of achieving this goal.
